# Supplementary material for: Identification of the ventral occipital visual field maps in the human brain
Source: F1000Res. 2017 Aug 21;6:1526. [Version 1] doi: 10.12688/f1000research.12364.1 (PMC5687318; doi:10.12688/f1000research.12364.1)
Supplement: Peer review history

# Peer review: Referee response for version 1

**DOI:** 10.5256/f1000research.13389.r27268

**Published:** 2017-11-06

**Authors:** Antony B. Morland

**Affiliations:** 1 Department of Psychology, York Neuroimaging Centre, University of York, York, UK

I rather like this short paper that reports on how to identify ventral visual field maps. Having had to attempt to do this on many occasions, sometimes with limited success, I find the paper very useful in giving evidence based guidance that could really help. The video and figures that support the paper are excellent and offer a resource for early career researchers to build up confidence in what can be a challenging task.

 One suggestion I have that might broaden the appeal of the study is to reflect a bit more on the relationship between the layout found in human and that in macaque. V4 is split between dorsal and ventral representations of quadrants, whereas the human data are often consistent with the forth (ventral) map being a representation of a hemifield. The authors touch on this early on, but having presented their data, they may reflect further on this in the Discussion.

**Minor points and suggestions**

*‘The multiple images for each subject were aligned and averaged. Averaging multiple anatomical images is desirable as it increases the contrast of the boundary between the grey and white matter, and therefore aids segmentation and the creation of an accurate cortical surface.’*

 I agree, but the authors might also point out that multiple, relatively short, acquisitions are also probably better than using longer acquisitions during which participant motion could work against benefits in contrast.

 I understood every word of the section that described the functional methods, but I would because I pretty much use the same methods. Are more details required for a naive reader?  

 Anatomical Figure (Figure 2) I think it would be good to use colour to mark the sulci/gyri and colour the labelling text in line with the sulcal colour. That way repeats of lettering can be avoided and less ambiguity between the location of text and the anatomical feature can be achieved.

 Section on calcarine sulcus. Perhaps a bit more text on the variants observed at the pole - there is a reasonably well-documented ‘Y’ configuration too, I think.

 Section 6.1 'The peripheral boundary is usually not identified with fMRI because the field of view that can be achieved during scanning is less than the field of view represented in the maps.' *I assume this refers the the size of the stimulus, not the FOV used in imaging, but some clarification is needed here.*

 'and each map is traced as far into the fovea as the resolution in the angle maps allow, usually one or two degrees from fixation'

*I think representation needs to be inserted here a couple of times e.g. ‘as far into the representation of the fovea’ and ‘usually at the representation of one or two degrees from fixation’*

I have read this submission. I believe that I have an appropriate level of expertise to confirm that it is of an acceptable scientific standard.

# Peer review: Referee response for version 1

**DOI:** 10.5256/f1000research.13389.r25204

**Published:** 2017-09-06

**Authors:** Geoffrey K. Aguirre

**Affiliations:** 1 Department of Neurology, University of Pennsylvania, Philadelphia, PA, USA

Winawer and Witthoft describe a standard procedure to define human visual area hV4 within retinotopic mapping data obtained using fMRI. The definition of the early, retinotopically organized visual areas is a crucial first step in many studies of the cortical organization for vision. While areas V1-V3 are readily identified within functional MRI mapping data, area hV4 is often challenging due to reduced signal strength, imaging artifacts, and the peculiar organization of this region.

 The paper documents and supports a supplementary video, which is primarily a presentation of the figures from the paper. This video, narrated in the even, velvet tones of the first author, serves as a very nice introduction to ventral retinotopic maps generally, and to the specific procedure of identifying the borders of area hV4. The video is a sufficiently important resource that I suggest that the authors mention it in the abstract.

 I have no concerns regarding the method or the conclusions of the paper. Instead, I have two things that I would have ideally liked to have seen discussed:

 First, for the purposes of this report, I think it is appropriate for the authors to assume that the reader has in hand a retinotopic map that is displayed on an inflated cortical surface. They are therefore justified in providing minimal treatment of the stimulus, MRI acquisition parameters, and initial analysis approach. I would have liked, however, some observations regarding choices one might make in these methods that impact the identification of ventral visual areas. For example, they might observe that retinotopic mapping need not extend into the far periphery to define hV4, as the region has a fairly compressed representation of the periphery. Similarly, it would be helpful to mention that different choices can be made in the content of the mapping stimulus that could enhance the responses from hV4. Finally, they might comment upon choices made in EPI vs. spin-echo imaging that might reduce the influence of the transverse venous sinus upon the hV4 functional data, or MR-V imaging techniques that are useful in the identification of this venous structure. These points could also be addressed in a brief paragraph in the discussion.

 Second, as the anatomical landmarks are an important guide for the identification of hV4, the authors could comment upon their variability, both within the volumetric space and within a spherical registration space of cortical topology. To my understanding, it is this variability (coupled with variations in structure-function relationships and measurement limitations) that drives the need for individual definition of this region.

 Finally, there is a typo in the introduction: the word "calcarine" is unnecessarily capitalized.

I have read this submission. I believe that I have an appropriate level of expertise to confirm that it is of an acceptable scientific standard.

# Peer review: Referee response for version 1

**DOI:** 10.5256/f1000research.13389.r25205

**Published:** 2017-08-23

**Authors:** Ben M. Harvey

**Affiliations:** 1 Experimental Psychology, Helmholtz Institute, Utrecht University, Utrecht, Netherlands

Winawer and Witthoft present a methodological paper describing the practical details that allow researchers to localize the human homologue of V4 (hV4) using fMRI visual field mapping. As described in the Abstract and Introduction, it has become very straightforward to define earlier visual field maps (V1-V3), but hV4 presents particular difficulties that prevented researchers even agreeing hV4’s location for many years. Studies by Winawer and Witthoft themselves addressed this disagreement. Other researchers in the field have typically learned to localize hV4 after demonstrations of the practical details from experienced colleagues. In most studies, many of these practical details are omitted for brevity, and because parts have been described elsewhere. It is becoming increasingly clear that visual field map definitions beyond V1-V3 are not as consistent as they should be across labs. So, the field will benefit from publication of more standardized, practical protocols like those described here to allow consistent localization of hV4. The approach described here is methodologically very strong and clearly described for an inexperienced researcher.

 Major points:

 The manuscript describes procedures for drawing V1-3 and hV4. However, the anterior boundary of hV4 is described in relation to VO1 (section 6.2.1), and procedures for drawing VO1 are not described. Furthermore, VO1 is fairly far anterior of hV4, so will often not be covered in a scan volume targeted at V1-3 and hV4. I don’t think it is wise to add procedures for drawing VO1-2, because these procedures rely on their relationship to hV4, making quite a circular description. Therefore, this section would benefit from rewriting without assuming that VO1 has been drawn already, i.e. based on the location of an area of maximum preferred eccentricity in an eccentricity gradient, rather than based on an eccentricity reversal to VO1.

 Procedures for drawing hV4 are described clearly, but assume the researcher already has good surface renderings and estimates of each voxel’s preferred visual field position. These earlier stages are extensive and complex, but the manuscript describes them only briefly and with reference to previous studies. I don’t believe an inexperienced researcher could work through these stages from the brief descriptions in the manuscript, so effectively the manuscript assumes the reader already has considerable experience. To make a more practical, step-by-step description of the complete procedure, a more complete description of normal segmentation and pRF modelling procedures would be valuable. However, I understand if the authors decide against including this, because it would change the focus of the manuscript considerably.

 Minor points:

 The discussion of differences to expect between subjects is helpful, but the resulting definition of hV4 includes the areas where draining veins introduce artefacts. The manuscript should make clear that subsequent analyses of responses in these areas will be strongly affected by these artefacts. Indeed, for many subsequent analyses these areas give a corrupted view of hV4’s responses, and I would certainly exclude these areas from most subsequent analyses. So, the manuscript should make clear that, while these voxels do lie in hV4 anatomically, it is often preferable to exclude them from our definition of hV4.

 In Figure 7, the iso-eccentricity line with the highest eccentricity is misleading. In this figure, all voxels with preferred eccentricity above 6˚ are labelled in blue. The line running through this blue area therefore links voxels with different eccentricities, all above 6˚.

 The parahippocampal gyrus is mentioned as a landmark in section 5.2, but not shown on Figure 2. Please add this to the figure.

 In section 6.1, the following phrase is ambiguous: “it may be possible to trace V2 and V3 in concentric ‘V’ shapes around V1.” This could be taken to mean that each of the dorsal and ventral quarterfields of V2 and V3 is V-shaped (i.e. ending at a point, outside the foveal confluence), when in fact there are bands passing through the foveal confluence (Schira *et al*, reference 9). Please re-write this phrase to make it clear that each ‘V’ is a whole hemifield map of V2 or V3.

I have read this submission. I believe that I have an appropriate level of expertise to confirm that it is of an acceptable scientific standard, however I have significant reservations, as outlined above.
